# The immune response after hypoxia-ischemia in a mouse model of preterm brain injury

**DOI:** 10.1186/s12974-014-0153-z

**Published:** 2014-09-05

**Authors:** Anna-Maj Albertsson, Dan Bi, Luqi Duan, Xiaoli Zhang, Jianmei W Leavenworth, Lili Qiao, Changlian Zhu, Susanna Cardell, Harvey Cantor, Henrik Hagberg, Carina Mallard, Xiaoyang Wang

**Affiliations:** Perinatal Center, Department of Neuroscience and Physiology, Sahlgrenska Academy, University of Gothenburg, Box 432, SE-405 30 Gothenburg, Sweden; Department of Pediatrics, The Third Affiliated Hospital of Zhengzhou University, Zhengzhou, China; Department of Cancer Immunology and AIDS, Dana-Farber Cancer Institute, Harvard Medical School, Boston, MA USA; Department of Microbiology and Immunobiology, Division of Immunology, Harvard Medical School, Boston, MA USA; Department of Pediatrics, Song Jiang Central Hospital, Shanghai, China; Center for Brain Repair and Rehabilitation, Department of Neuroscience and Physiology, Sahlgrenska Academy, University of Gothenburg, Gothenburg, Sweden; Department of Microbiology and Immunology, Institute of Biomedicine, Sahlgrenska Academy, University of Gothenburg, Gothenburg, Sweden; Department of Clinical Sciences, East Hospital, 416 85 Gothenburg, Sweden; Centre for the Developing Brain, Department of Perinatal Imaging and Health, King’s College London, St. Thomas’ Hospital, London, SE1 7EH UK

**Keywords:** Brain injury, Preterm, Hypoxia-ischemia, Immune response

## Abstract

**Background:**

Preterm brain injury consists primarily of periventricular leukomalacia accompanied by elements of gray-matter injury, and these injuries are associated with cerebral palsy and cognitive impairments. Inflammation is believed to be an important contributing factor to these injuries. The aim of this study was to examine the immune response in a postnatal day (PND) 5 mouse model of preterm brain injury induced by hypoxia-ischemia (HI) that is characterized by focal white and gray-matter injury.

**Methods:**

C57Bl/6 mice at PND 5 were subjected to unilateral HI induced by left carotid artery ligation and subsequent exposure to 10% O_2_ for 50 minutes, 70 minutes, or 80 minutes. At seven days post-HI, the white/gray-matter injury was examined. The immune responses in the brain after HI were examined at different time points after HI using RT-PCR and immunohistochemical staining.

**Results:**

HI for 70 minutes in PND 5 mice induced local white-matter injury with focal cortical injury and hippocampal atrophy, features that are similar to those seen in preterm brain injury in human infants. HI for 50 minutes resulted in a small percentage of animals being injured, and HI for 80 minutes produced extensive infarction in multiple brain areas. Various immune responses, including changes in transcription factors and cytokines that are associated with a T-helper (Th)1/Th17-type response, an increased number of CD4+ T-cells, and elevated levels of triggering receptor expressed on myeloid cells 2 (TREM-2) and its adaptor protein DNAX activation protein of 12 kDa (DAP12) were observed using the HI 70 minute preterm brain injury model.

**Conclusions:**

We have established a reproducible model of HI in PND 5 mice that produces consistent local white/gray-matter brain damage that is relevant to preterm brain injury in human infants. This model provides a useful tool for studying preterm brain injury. Both innate and adaptive immune responses are observed after HI, and these show a strong pro-inflammatory Th1/Th17-type bias. Such findings provide a critical foundation for future studies on the mechanism of preterm brain injury and suggest that blocking the Th1/Th17-type immune response might provide neuroprotection after preterm brain injury.

## Background

Prematurity is associated with an elevated risk of suffering from brain injuries, and these brain injuries are associated with impaired quality of life due to disorders such as cerebral palsy and behavioral, social, attentional, and cognitive deficits. The most common form of brain injury in preterm infants is generally thought to consist primarily of periventricular leukomalacia (PVL), a specific form of cerebral white-matter injury that is often accompanied by elements of gray-matter injury [1-3]. PVL can be either cystic necrotic or non-cystic. The cystic necrotic form is associated with the loss of all cellular elements, but the non-cystic lesions are more diffuse and cell-specific. The diffuse non-cystic lesions are the most common form of injury seen in preterm infants at present, and these primarily affect premyelinating oligodendrocytes (preOLs) leading to either loss of these cells or their inability to differentiate into mature oligodendrocytes, which results in cerebral hypomyelination [4-6].

Hypotensive episodes and hypoxic events, inflammation and infection [7-9], and tissue hypoxia arising from inflammation/infection are the major initiating factors of preterm brain injury. The ischemia-reperfusion injury process has been confirmed by the presence of infarctions in the arterial end and border zones of the periventricular white matter [10,11] and by the observation of pressure-passive circulation without autoregulatory function in newborn infants [12]. The most commonly used method for studying hypoxia-ischemia (HI)-induced brain damage in the neonatal brain is a rodent HI model referred to as the Vannucci model [13]. This model is very well characterized for mice and rats between postnatal day (PND) 7 and PND 10 [14-21], and adaptations in younger rodent pups including PND 3 to 6 rats [22] and PND 6 to 7 mice [23,24] have also been developed. The periventricular white matter in humans is highly susceptible to HI at gestational weeks 24 to 32 due to its high proportion of preOLs, and it is known that the number of preOLs is highest at PND 2 to 5 in rodents and that this number quickly declines after this developmental stage [25]. By PND 6–7, extensive oligodendrocyte (OL) maturation occurs in rodents and this coincides with the onset of early myelination [26]. Thus, white-matter vulnerability in the rodent at PND 2 to 5 would correspond to that in preterm human infants.

HI-induced injury to the immature brain has been found to induce the expression of various genes related to immune responses and inflammation, including macrophage and microglia-related genes, T-lymphocyte–related genes, and cytokines [27]. In addition, inflammatory mediators have been suggested to contribute to injury after HI in the immature brain [28] at a stage corresponding to human term and near-term infants. The immune system develops rapidly immediately after birth. For example, mice are not able to produce detectable levels of humoral antibodies in response to antigen until after one week of age, and the antigen-presenting dendritic cells do not reach normal levels as it is in adult rodent until one week after birth [29]. This indicates that the inflammatory response is likely to be very different during the first days after birth compared to one week later, and the differences are even more pronounced compared to older rodents [29]. However, the inflammatory and immune responses after HI brain injury in mice younger than PND 7 have not been characterized. The first aim of this study was to use the Vannucci model to find the duration of hypoxia that causes focal white/gray-matter injury in PND 5 mice, a developmental age in mice that corresponds to the preterm human infant. The second aim was to characterize the immune/inflammatory response after HI in this preterm injury model.

## Methods

### Animals

Pregnant C57BL/6 J mice were purchased from Charles River Laboratories (Sulzfeld, Germany) and these gave birth in the animal facility at the University of Gothenburg (Experimental Biomedicine, University of Gothenburg). The day of birth was defined as PND 1. Mice were housed with a 12-hour light/dark cycle, and free access to a standard laboratory chow diet (B&K, Solna, Sweden) and drinking water was provided. All animal experiments were approved by the Animal Ethical Committee of Gothenburg (Number 51/2012, 5/2013).

### Hypoxia-ischemia procedure

At PND 5, mice of both sexes were subjected to HI insult according to a method described previously with some modifications [13]. Briefly, mice were anesthetized with isoflurane (5.0% for induction and 1.5 to 3.0% for maintenance) in a 1:1 mixture of nitrous oxide and oxygen. The left common carotid artery was ligated, and the mice were returned to their cage and allowed to recover for one hour. The mice were then placed in an incubator perfused with a humidified gas mixture (10 ± 0.01% oxygen in nitrogen) at 36°C for 50 minutes, 70 minutes, or 80 minutes. After HI, the pups were returned to their dam until being killed. The combination of artery ligation and hypoxia resulted in injury only in the hemisphere ipsilateral to the artery ligation (the left hemisphere), while no injury was produced in the contralateral hemisphere (the right hemisphere). PND 9 mice were subjected to hypoxia for 50 minutes to produce a comparable degree of injury in the hippocampus as seen after 70 minutes HI in the PND 5 mice [30].

### Assessment of brain damage

Mice were deeply anesthetized and perfused intracardially with saline and 5% buffered formaldehyde (Histofix; Histolab Products AB, Gothenburg, Sweden). The brains were dissected, paraffin-embedded, and cut into 10-μm coronal sections throughout the whole brain. Immunohistochemical staining for microtubule-associated protein 2 (MAP-2) and myelin basic protein (MBP) was performed on every 50th section.

The extent of white- and gray-matter injury was analyzed by quantitative measurements of the injury area (at 4× magnification) using Micro Image version 4.0 (Micro-macro AB, Gothenburg, Sweden). The MAP-2-positive or MBP-positive area (remaining tissue) was measured in each hemisphere as previously described [31,32], and total tissue loss was calculated as:

$$ \left(\mathrm{Contralateral}\ \mathrm{hemisphere}\;\hbox{--}\;\mathrm{ipsilateral}\ \mathrm{hemisphere}\right)\;/\;\left.\mathrm{Contralateral}\ \mathrm{hemisphere}\right)\times 100\% $$

Coronal sections at the hippocampal level were used for all other staining. Thionin/acid fuchsin staining was performed as described previously [33]. Briefly, the sections were dipped in 1% thionin/toluidin solution, dipped into acid fuchsin solution, and dehydrated in a gradient of ethanol before mounting.

### Immunohistochemical staining

After antigen recovery and blocking, the following primary antibodies were used: mouse anti-MAP-2 (clone HM-2, Sigma-Aldrich, Stockholm, Sweden), mouse anti-MBP (SMI94, Covance, NJ, USA), goat anti-triggering receptor expressed on myeloid cells-2 (TREM-2, clone G-18, Santa Cruz Biotechnology, Santa Cruz, CA, USA), rabbit anti-ionized calcium binding-adapter molecule 1 (Iba-1, Wako Chemicals, Richmond, VA, USA), and mouse anti-cluster of differentiation 4 (CD4, Vector Laboratories, Burlingame, CA, USA). After incubation with the appropriate biotinylated secondary antibody (Vector Laboratories, Burlingame, CA, USA), visualization was performed using Vectastain ABC Elite with 3,3′-diaminobenzidine (DAB) enhanced with ammonium nickel sulfate, beta-D glucose, ammonium chloride, and beta-glucose oxidase (all from Sigma-Aldrich, Stockholm, Sweden).

For fluorescent triple staining, the sections were incubated with goat anti-TREM-2, mouse anti-DNAX activating protein 12 antibody (DAP12, clone B-2, Santa Cruz Biotechnology, Santa Cruz, CA, USA) and rabbit anti-Iba-1. The sections were then incubated with the appropriate Alexa Fluor 594 donkey-anti-rabbit or Alexa Fluor 488 donkey-anti-mouse (Molecular Probes, Leiden, Netherlands) secondary antibody in PBS, and a coverslip was mounted using ProLong Gold antifade reagent with 4,6-diamidino-2-phenylindole (DAPI) (Life Technologies, Carlsbad, CA, USA).

### RT-PCR

Changes in cytokine gene expression in the brain at 6 hours, 24 hours, or 7 days after HI in PND 5 and control mice were analyzed by RT-PCR. After a brief intracardial perfusion with saline, the brains were rapidly dissected, divided into ipsilateral and contralateral hemispheres, snap frozen, and stored at −80°C until use.

The brain tissue was homogenized with Qiazol lysis reagent homogenizer (Qiagen, Sollentuna, Sweden), and total RNA was extracted using the RNeasy Lipid Tissue Mini Kit (Qiagen, Sollentuna, Sweden). RNA was measured with a spectrophotometer at 260 nm absorbance. cDNA was synthesized using a QuantiTect Reverse Transcription Kit (Qiagen, Sollentuna, Sweden) following the manufacturer’s instructions, and each sample was run in duplicate. The following primers were used: RAR-related orphan receptor gamma (RORγt, QT00197722), T-bet (QT00129822), GATA binding protein 3 (GATA3, QT00170828), forkhead box P3 (Foxp3, QT00138369), IL-4 (QT00160678), IL-5 (QT00099715), IL-6 (QT00098875), IL-10 (QT00106169), IL-12a (p35) (QT01048334), IL-13 (QT02423449), IL-17α (QT00103278), IL-21 (QT01758036), IL-22 (QT00128324), IL-23a (QT01663613), transforming growth factor beta (TGF-β, QT01038821), IFN-γ (QT00145250), B- and T-lymphocyte attenuator (BTLA, QT00117635), CXCR5 (QT00253449), CXCL13 (QT00107919), inducible T-cell co-stimulator (ICOS, QT02253552), and 18S ribosomal RNA (QT01036875) (all from Qiagen, Sollentuna, Sweden). Melting curve analysis was performed to ensure that only one PCR product had been produced. A standard curve was generated for quantification and for estimating amplification efficiency using increasing concentrations of cDNA, and the amplification transcripts were quantified with the relative standard curve and normalized to the 18S ribosomal RNA reference gene.

### Statistics

The Statistical Package for the Social Sciences (SPSS Inc., Chicago, IL, USA) v19.0 software package was used for all analyses. Comparisons between groups were performed by Student’s *t*-test, and data with unequal variance were compared with the Mann-Whitney *U*-test. Analysis of variance followed by the LSD *post-hoc* test was used for comparison of data from more than two groups. *P* < 0.05 was considered statistically significant.

## Results

### White/gray-matter injury after HI in PND 5 mice

**Figure 1:**
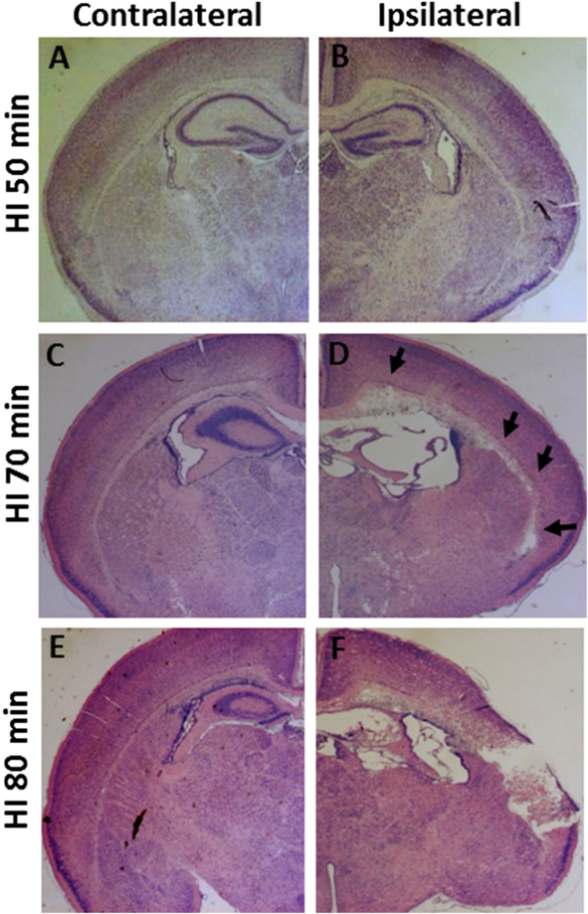
**Representative photomicrographs of thionin/acid fuchsin-stained brain sections.** It shows different degrees of injury in the ipsilateral hemisphere at 3 days after HI with 50 minutes (**A**, **B**, n = 12), 70 minutes (**C**, **D**, n = 14), or 80 minutes (**E**, **F**, n = 16) of hypoxia. Arrows in **(D)** indicate focal subcortical white-matter injury in the ipsilateral hemisphere of the HI 70-minute mouse brain.

**Figure 2:**
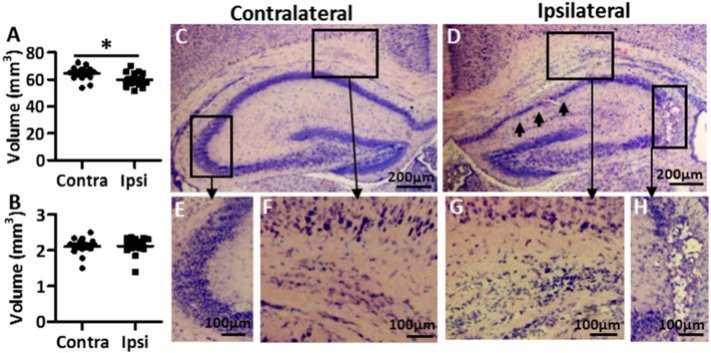
**Brain injury after 50-minute hypoxia-ischemia (HI) insult.** Dot graphs show the volume difference between the ipsilateral and contralateral hemispheres in gray matter **(A)** and white matter **(B)** at 7 days after 50-minute HI in PND 5 mice (n = 16). Abbreviations: Contra: contralateral hemisphere; Ipsi: ipsilateral hemisphere. *P < 0.05 by Student’s unpaired *t*-test. Data are presented as mean ± SEM. **(C-H)** Representative photomicrographs of thionin/acid fuchsin-stained sections show diffuse injury in the subcortical white matter area **(D and G)** and focal granular layer loss in the hippocampus CA3 area **(D and H)** that is only observed in the ipsilateral hemisphere and not in the contralateral hemisphere **(C, E, and F)** at 3 days after HI in PND 5 mice. Arrows in **(D)** indicate focal injury in the CA1 granular layer of the hippocampus in the ipsilateral hemisphere.

To determine the duration of hypoxia in PND 5 mice that produces mild and focal white/gray-matter injury, we tested hypoxia durations of 50 minutes, 70 minutes, and 80 minutes in combination with left carotid artery ligation using the Vannucci model. Mice were killed three or seven days after HI to evaluate the extent of brain injury. All pups tolerated HI well and none of the pups died during or soon after the HI insult. Thionin/acid fuchsin staining showed that after 50 minutes of hypoxia only 3/12 (25%) of the mice had suffered visible brain injury (Table 1). The injury was mostly found in the hippocampus and was characterized by loss of neurons in the CA1 to CA3 areas in the cerebral hemisphere ipsilateral to the carotid ligation (Table 1, Figure 1B, arrows in Figure 2D and 2H). No injury was found in the contralateral hemisphere (Figure 1A and Figure 2C and 2E). Only 2/12 (17%) of the mice displayed mild local white-matter tissue disruption in the ipsilateral hemisphere (Figure 2D and 2G) in comparison to the contralateral hemisphere (Figure 1A and Figure 2C and 2F).

**Table 1:** **Brain injury with different durations of hypoxia-ischemia (HI) in postnatal day (PND) 5 mice**

**Hypoxia time**	**Percent of injured mice**	**CX injury**	**Hipp. injury**	**Subcortical WM injury**
50 minutes	25%	No	Loss of granular cells in the CA areas	Localized and diffuse
70 minutes	93%	selective neuronal injury	Tissue structure disruptions	WM diffuse injury and tissue structure disruptions
80 minutes	88%	Infarction	Infarction	Infarction

*Abbreviations*: *CA* cornu ammonis, *CX* cortex *HI* hypoxia-ischemia, *Hipp* hippocampus, *PND* postnatal day, *WM* white matter.

**Figure 3:**
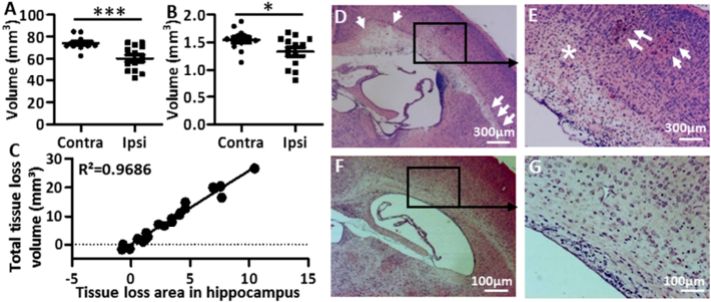
**Brain injury after 70-minute hypoxia-ischemia (HI) insult.** Dot graphs showing the volume difference between the contralateral and ipsilateral hemispheres in gray matter **(A)** and white matter **(B)** at 7 days after 70-minute HI in PND 5 mice (n = 13). *P < 0.05, ***P < 0.001 using Student’s unpaired *t*-test. Data are presented as mean ± SEM. Abbreviations: Contra: contralateral hemisphere; Ipsi: ipsilateral hemisphere. **(C)** The simple linear regression analysis shows the linear correlation between the two evaluation methods. **(D-G)** Representative photomicrographs of H&E-stained sections show focal injury in the subcortical white matter in the ipsilateral hemispheres at 3 days after HI in PND 5 mice **(D and E)** but not in PND 9 mice **(F and G)** with a similar degree of injury. Arrows in **(D)** show subcortical white-matter injury. Arrows and star in **(E)** show focal gray-matter injury in the ipsilateral hemisphere of the PND 5 HI mouse brain.

When the duration of hypoxia was extended to 70 minutes, 13/14 (93%) of the mice were injured (Table 1). The injury was characterized by hippocampal injury in combination with focal white-matter injury (arrows in Figure 1D, and Figure 3D) as well as small areas of focal cortical injury in the ipsilateral hemisphere (star and arrows in Figure 3E). Only 1/14 (7.1%) of the mice showed cortical infarction. Notably, the subcortical white matter area in the PND 5 mice showed significantly greater vulnerability compared with the surrounding striatum and cortex in the same area (arrows in Figure 1D and Figure 3D), which was not seen in PND 9 HI mice (Figure 3F and 3G).

In pups subjected to 80 minutes of HI, 14/16 (88%) of the mice were injured and 6/16 (37.5%) of the mice displayed extensive infarction in the cortex, hippocampus, white matter, and thalamus in the ipsilateral hemisphere (Table 1 and Figure 1F). No injury was observed in the contralateral hemisphere (Figure 1E).

To evaluate the effect of HI on myelination and gray-matter injury, MBP and MAP-2 immunostaining was performed at PND 12 (7 days after HI), which is the age when myelin begins to be detectable in mice [34]. After 50 minutes of HI, there was a significant decrease in gray-matter volume (Figure 2A) in the ipsilateral hemisphere compared with the contralateral hemisphere. There was no significant difference observed in terms of myelination (Figure 2B) between the two brain hemispheres.

In pups exposed to 70 minutes of HI, both the gray-matter volume (Figure 3A) and the white-matter volume (Figure 3B) in the ipsilateral hemisphere were significantly reduced compared with the contralateral hemisphere at 7 days post-HI (PND 12). A simple linear regression analysis comparing the total brain tissue loss (volume) with the tissue loss in one representative brain section (area) from the hippocampus level showed a significant positive linear correlation between the two methods (Figure 3C, *P* < 0.001, R^2^ = 0.9686).

**Figure 4:**
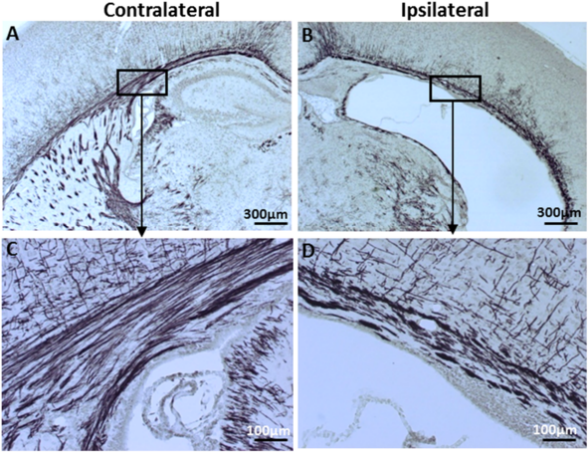
**Impaired myelin structure at 7 days after 70-minute hypoxia-ischemia (HI).** Representative photomicrographs of myelin basic protein (MBP)-stained brain sections at the hippocampal level show impaired myelin development and disrupted myelin structure in the subcortical white matter of the ipsilateral hemisphere **(B and D)** compared with the contralateral hemisphere **(A and C)**.

At 7 days after HI (PND 12) when myelination was formed and was visible by MBP staining, the subcortical white matter displayed abnormal myelin structure (Figure 4B and 4D) in the ipsilateral hemisphere compared to the normal morphology of the subcortical white matter in the contralateral hemisphere (Figure 4A and 4C). Quantification of MBP-positive immunostaining in the subcortical white matter area demonstrated that the subcortical white matter volume loss in the ipsilateral hemisphere for the 70 minutes HI group was 14.0 ± 4.2%, whereas no white matter loss was seen in the 50 minutes HI group. No gender differences were found for any of the hypoxia durations in this study.

We found that 70 minutes of HI results in focal diffuse white-matter injury combined with hippocampus injury, small areas of focal cortical injury, and a low frequency of cortical infarction. We concluded, therefore, that 70 minutes is the optimal duration of HI to induce brain injuries in PND 5 mice, and this model was used in the subsequent characterization of the inflammatory/immune responses after HI insults.

### Increased DAP12 and TREM-2 expression after HI

Triggering receptors expressed on myeloid cells (TREMs) are innate immune receptors that play an important role in fine-tuning the inflammatory response by altering the innate immune response. DNAX activation protein 12 (DAP12) is a transmembrane signaling adaptor protein that forms a molecular complex with TREM-2. TREM-2 is expressed on the cell membrane of monocyte-derived dendritic cells, osteoclasts, and microglia. Activation of DAP12 and TREM-2 plays an important role in demyelination disease in humans, and this led us to examine the possible role of this pathway in preterm brain injury, which is characterized by extensive white-matter injury.

**Figure 5:**
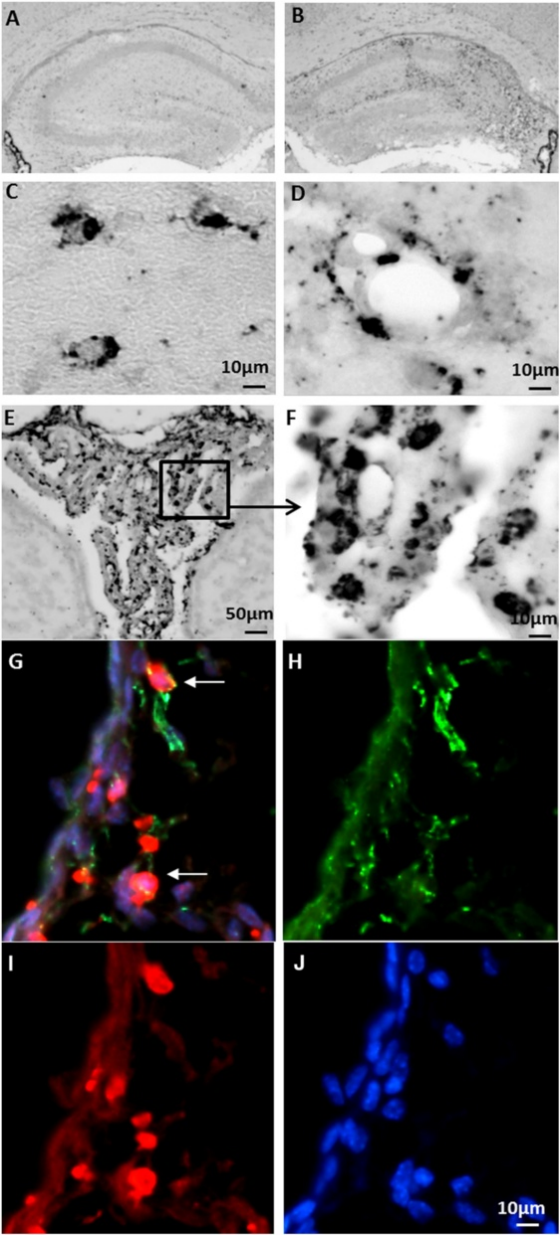
**TREM-2 expression after 70-minute hypoxia-ischemia (HI) insult.** Representative photomicrographs show TREM-2^+^ immunohistochemical staining in the contralateral **(A)** and ipsilateral **(B)** hemispheres at 24 hours after HI. **(C and D)** TREM-2^+^ cells in the hippocampus in the brain parenchyma **(C)** and blood vessels **(D)**. **(E)** TREM-2^+^ cells in the choroid plexus at 24 hours after HI. **(F)** A higher magnification of TREM-2^+^ staining. **(G-L)** TREM-2 staining **(H)** co-localizes (**G**, arrows) with microglia marker Iba-1 **(I)** in the choroid plexus at 24 hours after HI. **(J)** DAPI nuclear staining.

Brain sections from the hippocampal level were analyzed by immunohistochemistry at 24 hours, 3 days, and 7 days after 70-minute HI at PND 5. TREM-2 expression in the brain was increased in the ipsilateral hemisphere after HI with maximum expression seen at 24 hours after HI (Figure 5A and 5B). The increased TREM-2 expression at 24 hours after HI was mostly observed in the ipsilateral hippocampus, subcortical white matter, and white matter/thalamus (Figure 5B, 5C, and 5D). TREM-2 expression was also found in the meninges and the choroid plexus structure in the lateral and third ventricle (Figure 5E and 5F). The TREM-2^+^ cells were located both within the brain parenchyma (Figure 5C) and along the blood vessels (Figure 5D). The triple fluorescent staining of TREM-2 with microglia marker Iba-1 and DAPI nuclear staining indicated that some TREM-2^+^ cells are microglia (Figure 5G-J). The TREM-2 expression in the lateral ventricles displayed more TREM-2^+^ staining in the ipsilateral lateral ventricle compared to the contralateral ventricle, and the expression was higher in the choroid plexus of the third ventricle at 24 hours after HI compared to age-matched undamaged controls. However, the TREM-2^+^ staining in the meninges did not differ between the two hemispheres.

**Figure 6:**
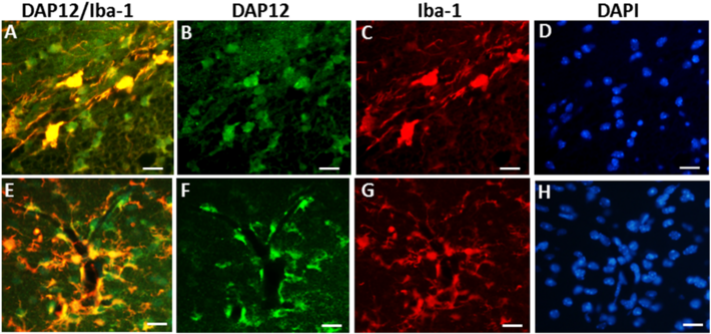
**DAP12 expression after a 70-minute hypoxia-ischemia (HI) insult.** Representative photomicrographs show expression of DAP12 (green) and its co-localization with the microglia marker Iba-1 (red) after HI. DAP12 and Iba-1 double-positive cells were found in the dentate gyrus **(A-D)** at 24 hours after HI, and in the proximity of blood vessels in the CA1 area of the hippocampus **(E-H)** at 3 days after HI.

The DAP12 expression in the brain after 70 minutes HI at PND 5 was evaluated by immunofluorescence staining at 24 hours, 3 days, and 7 days after HI. The expression of DAP12 was found in the injured area in the subcortical white matter area, the hippocampus, the periventricular area, and the meningeal areas in the ipsilateral hemisphere. Triple immunofluorescence staining for DAP12, the microglia marker Iba-1 and DAPI showed that DAP12 and Iba-1 double-positive cells were found in the ipsilateral periventricular area at 24 hours after HI (Figure 6A-6D). Iba-1 and DAP12 double-positive cells were also detected close to blood vessels in the ipsilateral hippocampus (Figure 6E-6H) and in the meningeal area at three days after HI.

### The immune cell response after HI in PND 5 mice

**Figure 7:**
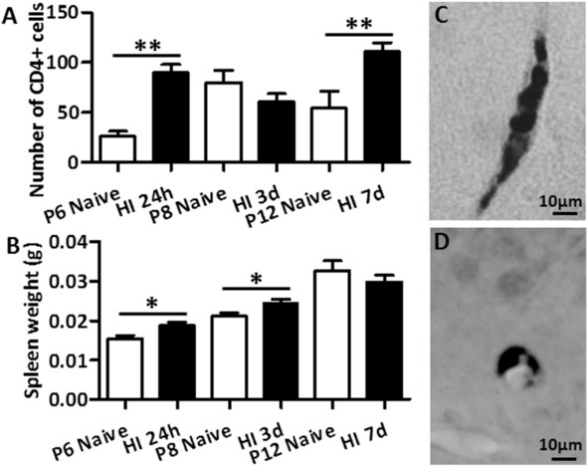
**CD4** ^**+**^ **T-cells in the mouse brain after hypoxia-ischemia (HI) in postnatal day (PND) 5 mice.** The total number of CD4^+^ cells in blood vessels and in tissue from both of the brain hemispheres from a section at the hippocampus level **(A)** and spleen weight **(B)** in uninjured control (Cont) mice versus HI mice at different time points after HI. *P < 0.05, **P < 0.01, ***P < 0.001 using Student’s unpaired *t*-test. Data are presented as mean ± SEM. **(C and D)** Representative immunostainings show CD4^+^ cells in blood vessels in the ipsilateral subcortical white matter **(C)** and ipsilateral hippocampus CA1 area **(D)** at seven days post-HI.

To examine the possible infiltration of immune cells into the brain after HI, we used immunohistochemical staining with an antibody against CD4. Indeed, the number of CD4^+^ cells in the brain increased at 24 hours and 7 days after HI compared with age-matched undamaged control mice (Figure 7A). Most of the CD4^+^ cells were seen in blood vessels, and only a few were seen in the brain tissue (Figure 7C and 7D). Because it is one of the most important organs of the immune system, spleen weight was measured at 24 hours, 3 days, and 7 days after HI and in undamaged control mice. A significant increase in spleen weight was seen at 24 hours and 3 days, but not at 7 days, after HI compared to controls (Figure 7B) suggesting that an HI-induced global inflammatory response might increase the trafficking of immune cells, including CD4^+^ T-cells, into the injured brain during the early phase of inflammation.

### Different canonical types of cytokine responses after HI in PND 5 mice

**Figure 8:**
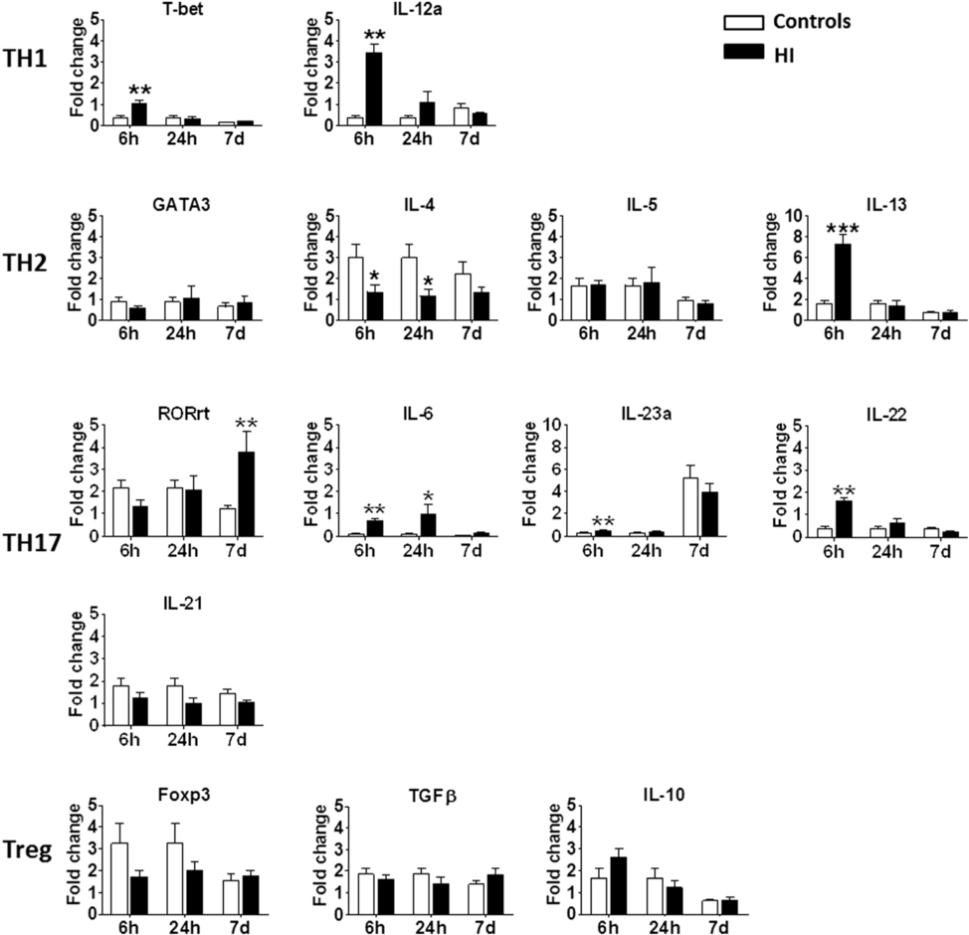
**The canonical types of cytokine and transcription factor gene expression in the brain after hypoxia-ischemia (HI).** *P < 0.05, **P < 0.01, ***P < 0.001 using Student’s unpaired *t*-test. Data are presented as mean ± SEM. N = 5 to 8/group. White bars: undamaged controls; Black bars: post-HI ipsilateral hemisphere. Abbreviations: Th: T-helper cells; Treg: T-regulatory cells.

To further evaluate the inflammatory/immune response in the HI-induced preterm brain injury model described above, brains were harvested at 6 hours, 24 hours, and 7 days after HI and from uninjured age-matched control mice. The expression of several cytokine mRNAs was altered in the ipsilateral brain hemisphere after HI (Figure 8) but there were no significant changes in the contralateral brain hemispheres after HI.

We first investigated whether the transcription of immunity-related genes, including those related to CD4+ T-helper (Th) cells, were affected by HI. At six hours after HI, there was a significant up-regulation of the mRNAs for the Th type 1 (Th1) cell transcription factor T-bet and the Th1 cell-promoting cytokine IL-12a in the brain (Figure 8). IFN-γ - the signature cytokine secreted from Th1 cells - was not detectable in the brain after HI in PND 5 mice.

The Th2 transcription factor GATA3 was not affected by HI, but the IL-4 cytokine, which promotes the differentiation of Th2 cells, was significantly down-regulated at 6 hours and 24 hours after HI. IL-13, a cytokine produced mainly by Th2 cells, was significantly up-regulated at 6 hours after HI, but no changes were observed for IL-5 (Figure 8).

For the Th17-type cytokine response, the transcription factor RORγt was significantly increased at 7 days after HI. The expression of the Th17-promoting cytokine IL-6 was significantly increased at 6 hours and 24 hours after HI. Cytokine IL-22, which is secreted from Th17 cells, was significantly increased at 6 hours after HI. IL-17A was not detected in the brain in the normal control animals at any of the time points analyzed.

For the T-regulatory (Treg) type of response, the expression of the Treg transcription factor Foxp3 was significantly decreased at 6 hours and 24 hours after HI. However, there was no change in levels of the Treg-promoting cytokine TGF-β or the Treg-secreted cytokine IL-10.

## Discussion

We have established a novel PND 5 mouse preterm brain injury model to show that both the innate and adaptive immune responses are activated after HI at this age. This model has allowed us for the first time to examine the responses of different subsets of Th cells in a neonatal HI model, and we found that HI induced a primarily Th1/Th17-type immune response in the neonatal mice.

Fifty minutes of HI produced mild injury localized in the white and gray matter, and this degree of injury is similar to that previously reported in PND 6 HI mice [23]. However, only 25% of the mice were damaged and most of the pups did not display any signs of injury. Furthermore, no significant differences were seen in the white-matter volume between the ipsilateral and contralateral hemispheres, and this limits the usefulness of the 50-minute HI exposure in neuroprotection studies. When the HI duration was extended to 80 minutes, the injury in the ipsilateral hemisphere was very extensive and included widely distributed infarction of the hippocampus, cortex, and thalamus that is rarely seen in preterm infants.

The model using 70 minutes of HI produced diffuse white-matter injury combined with focal neuronal loss in the cerebral cortex and hippocampus injury (Table 1). Indeed, at 7 days after HI (PND 12), the time when myelination becomes detectable in mice, we found impaired myelination in the subcortical white matter of the ipsilateral hemisphere (Figure 4B and 4D). Seventy minutes of HI significantly reduced both white- and gray-matter volume in 93% of the pups (Figure 3A and B), and this makes the model reliable and suitable for neuroprotection studies. Interestingly, we found that the degree of white-matter vulnerability after 70 minutes of HI at PND 5 (Figure 3D and 3E) was more obvious compared to that seen at PND 9 in mice (Figure 3F and 3G). This observation further confirms that PND 5 is within the time window when the white matter in the brain is more vulnerable to injury [35].

TREM-2 is expressed on the cell membrane of monocyte-derived dendritic cells, osteoclasts, and microglia and is associated with the signaling molecule DAP12 [36,37]. DAP12 in turn plays a signal transduction role in dendritic cells and macrophages and is considered to be both pro- and anti-inflammatory [38]. Apart from their function in the inflammatory response, DAP12 and TREM-2 clearly play an important role in white-matter disease and oligodendrocyte pathology. Mutations in TREM-2 and DAP12 cause sclerotic lesions in the white matter of the central nervous system (CNS) in human polycystic lipomembranous osteodysplasia with sclerosing leukoencephalopathy (PLOSL)/Nasu-Hakola disease [39,40] and cause hypomyelinosis in mice [41]. DAP12^+^ and TREM-2^+^ microglia/macrophages surround myelinating oligodendrocytes at PND 10 [42]. TREM-2 is expressed by microglia in the CNS both during normal development in mice [43] and in experimental autoimmune encephalomyelitis (EAE) - a mouse model of multiple sclerosis (a white-matter disease) [44,45]. Blockage of TREM-2 signaling using an anti-TREM-2 antibody increases the severity and progression of EAE [44].

We found increased expression of TREM-2 and DAP12 in the brain after HI with maximum expression at 24 hours after HI in the injured areas of the hippocampus and the white matter. DAP12 was co-expressed with microglia in the brain parenchyma, and this agrees with previous findings in the normal neonatal mouse brain [43] and in the rodent model of HI that is more representative of the type of brain injury seen in full-term infants [27]. The expression of DAP12 is found not only in injured areas but also in the meninges. Together with the finding that TREM-2 is also expressed in the choroid plexus and along the blood vessels, this indicates that the TREM and DAP12 pathway might serve as a sensor/communicator at the interface between the periphery and the CNS and might participate in the development of white-matter injury in preterm infants.

The accumulation of TREM-2, DAP12, and CD4^+^ T-cells in the meninges and choroid plexus and adherence to blood vessels might also have implications for the status of cerebrovascular reactivity. Similarly, another recent report showed that TREM-2 was expressed by some microglia/macrophages inside and around the blood vessels of the hippocampal fissure in PND 1 mouse brains, and such expression diminished with age [43]. The vasculature of the blood-brain barrier (BBB) protects the CNS from the systemic circulation by restricting entry of unwanted molecules and immune cells into the brain. Reports have shown that TREM-2 knockout mice have reduced invasion of CD3^+^ T-cells [46] through yet unknown mechanisms. Together with our finding that TREM-2 is located close to the blood vessels in the choroid plexus, this indicates that TREM-2 might actively participate in the invasion of peripheral T-cells into the brain through the choroid plexus and BBB by either modulating the BBB permeability in the brain or directly regulating the blood flow.

T-cells have been observed infiltrating the brain in the infarction area in animal models of stroke, and it is now widely accepted that T-cells play a role in both ischemic brain injury (stroke) and white-matter injury (multiple sclerosis) in both adult animal models and humans [47,48]. For example, T-cell-deficient mice exhibit significant reductions in infarct volume [49,50] in rodent models of HI brain injury. T-cells and other peripheral immune cells were found to infiltrate into the neonatal rat brain and to remain there for hours to months following HI [51-53].

In this study, we found that there were low, yet significantly increased, numbers of CD4^+^ cells in the brain after HI at PND 5 and that this was accompanied by changes in spleen weight, one of the most important organs in the immune system. At the time points examined in the present study, which are all relatively early for the adaptive immune response, the CD4^+^ T-cells are mostly located adhered to the blood vessels in the brain instead of in the brain parenchyma. This might well indicate that CD4^+^ cells are beginning to infiltrate into the brain at a relatively early stage in the adaptive immune response in addition to other cell types that are also involved in the early immune response in the brain.

Mammalian immune responses are often grouped into type 1 and type 2 responses [54]. These responses differ broadly in their mechanisms of induction as well as their innate cell types, cytokines, effector molecules, and Th types. The classical type 1 and type 2 responses are mediated by TCRαβ CD4^+^ Th cells and are thus generally referred to as Th1 and Th2 responses, respectively. It is now clear that many innate cell types such as γδ T-cells and innate lymphoid cells (ILCs) broadly parallel the known CD4^+^ Th cell subsets in terms of their signature cytokine secretion profiles. An overlapping series of transcription factors is also used to drive the differentiation of the various ILC and Th cell subsets. The results of our experiments show that HI leads to an imbalance between Th1/Th17-type responses and Th2/Treg-type responses, and these differences might be due to the recruitment and proliferation of different innate-type immune cells.

Type 1 immune responses are usually pro-inflammatory and are mediated by the type 1 cytokines such as IFN-γ, TNF-α, IL-6, and IL-17 [55]. In contrast, type 2 responses have immune-modulating functions and mediate allergic inflammatory diseases such as asthma, allergic rhinitis, and atopic dermatitis. Th2 cells regulate type 2 responses through the secretion of various type 2 cytokines, including IL-4, IL-5, IL-9, and IL-13. For many years, neonates have been considered to be immune deficient. It is now clear, however, that the newborn is capable of raising an immune response. Neonatal mice that have primary immune responses to certain foreign antigens normally mount Th2-type-dominant secondary responses when re-exposed to the same antigens. However, under certain pathogenic infections or after immunization with certain adjuvants that promote strong Th1-cell responses, such as DNA vaccines or oligonucleotides containing CPG motifs**,** neonates develop Th1-type responses that often induce the onset of diseases [54,56,57]. In HI-induced brain injury in neonatal mice, we observed an increased expression of Th1/Th17-related transcription factors that may reflect a dominant Th1-type response that could contribute to the brain injury. Indeed, high expression of Th1 and Th17-type cytokines has been found in PVL patients [58-60], and inhibition of Th17-type cytokines reduces brain injury in a neonatal mouse model [61]. Moreover, Th17 lymphocytes traffic to the CNS [62] and participate in the pathogenesis of CNS inflammatory demyelination disease [63]. These indicate that different types of immune responses might play different roles in preterm brain injury.

In this study, we examined different types of immune responses by examining the stimulatory cytokines that are present in the microenvironment during activation, transcription factors that prime naive precursor cells for differentiation, and the signature cytokine profiles for each type of response. We observed significantly increased levels of Th1-related (T-bet, IL-12a) and Th17-related (RORγt, IL-6, IL-23, and IL-22) transcription factors and cytokines after HI, and most Th2/Treg-related transcription factors and cytokines were either decreased (IL-4) or unchanged (GATA3, IL-5, Foxp3, TGFβ, and IL-10). Notably, the signature cytokine for Th17-related response (IL-17A) was not detectable in the neonatal mouse brain in the current study. We carefully analyzed the IL-17A expression in normal neonatal mice, but could not detect any mRNA under our experimental conditions. However, analysis of IL-17A expression in the present study focused on the early time points within 7 days post-HI when the adaptive immune responses are not initiated, and the early Th1/Th17-type response in the brain after HI might be due to innate-type cell types, for example, γδ T-cells and ILCs. Possibly, IL-17A could be expressed at later time points after HI when massive infiltration of T-cells into the CNS starts, since unlike its other analogs (that is, IL-17F), IL-17A is produced mainly in T-cells [64]. For the Th2-type response, we did find significantly increased levels of the Th2-type cytokine IL-13 in the brain at 6 hours after HI. IL-13 is a mediator of allergic inflammation and disease, and in the brain IL-13 has been found to induce oxidative stress and contribute to the death of activated microglia [65,66]. In general, it seems that the physiological Th2/Treg-type bias is lost in preterm brain injury after HI in mice and that the balance is instead skewed towards a type 1 (Th1/Th17) immune response (Figure 8).

Altogether, these results indicate that HI triggers a robust immune response in the brain that is characterized by an imbalance between Th1/Th17- and Th2/Treg-type responses. This imbalance occurs early in the immune response and is probably caused by an innate type of response as indicated by the increased yet low number of CD4^+^ cells mostly located in the blood vessels.

## Conclusions

Cerebral palsy is the most common severe neurological disability in children worldwide and is a heavy burden on those afflicted, their families, and society. Preterm brain injury is a major cause of cerebral palsy and there are currently no neuroprotective therapies available for premature infants. Inflammation plays a critical role in the development of preterm brain injury, and T-cells have been suggested to play essential roles in the response to ischemic brain injury [47,48,51,52]. Therefore, targeting the inflammation-specific immune cell population/pathways [9,67,68] might represent a new hope for the prevention and treatment of preterm brain injury. In the present study, we have established a reproducible PND 5 mouse model of HI-induced preterm brain injury, and we have further characterized the innate and adaptive immune responses and found the involvement of a Th1/Th17-type immune response after HI-induced preterm brain injury in the established model. This model will provide a powerful tool to study preterm brain injury mechanisms and to investigate possible neuroprotectants with the goal of discovering treatment strategies for preterm brain injury. The immune response observed in this model will provide a solid foundation for continued studies of the contribution of the neonatal immune system to preterm brain injury and provide evidence for neuroprotection by targeting specific inflammatory cascades after preterm brain injury.

## Abbreviations

BBB: blood-brain barrier
BTLA: B- and T-lymphocyte attenuator
CNS: central nervous system
DAB: 3,3′-diaminobenzidine
DAPI: 4,6-diamidino-2-phenylindole
DAP12: DNAX activation protein of 12 kDa
EAE: experimental autoimmune encephalomyelitis
Foxp3: forkhead box P3
GATA3: GATA binding protein 3
H&E: hematoxylin and eosin
HI: hypoxia-ischemia
IFN: interferon
IL: interleukin
ILCs: innate lymphoid cells
MAP-2: microtubule-associated protein 2
MBP: myelin basic protein
OL: oligodendrocyte
PCR: polymerase chain reaction
PLOSL: polycystic lipomembranous osteodysplasia with sclerosing leukoencephalopathy
PND: postnatal day
preOLs: premyelinating oligodendrocytes
PVL: periventricular leukomalacia
RORγt: RAR-related orphan receptor gamma
TGF-β: transforming growth factor beta
Th: T-helper cell
TNF: tumor necrosis factor
Treg: T-regulatory
TREM-2: triggering receptor expressed on myeloid cells 2
